# The Identification of Potential Treatment Targets to Reduce the Risk of Obesity‐Related Complications: A Step Toward a Treat‐to‐Target Approach in Obesity Management

**DOI:** 10.1002/osp4.70094

**Published:** 2025-11-05

**Authors:** Luca Busetto, Volker Schnecke, Maria Overvad, Silvia Capucci, Ricardo Reynoso, Rafael Bravo, Abd A. Tahrani, Camilla S. Morgen

**Affiliations:** ^1^ Department of Medicine University of Padova Padova Italy; ^2^ Novo Nordisk A/S Søborg Denmark; ^3^ Novo Nordisk Health Care Zürich Switzerland; ^4^ Metabolism and Systems Science University of Birmingham Birmingham UK

**Keywords:** cohort study, database research, obesity care, observational, primary care

## Abstract

**Aims:**

Obesity‐related complications (ORCs) are associated with substantial health and economic burdens. Although treatment targets are routinely used for other chronic conditions, none currently exist in obesity management. We aimed to identify an adiposity measure that indicates a reduced risk of four ORCs.

**Materials and Methods:**

This population‐based cohort study of patients aged 18–60 years used data from the UK Clinical Practice Research Datalink Aurum. Associations between absolute 10‐year ORC risk and baseline body mass index (BMI), waist–height ratio (WHtR), and changes in adiposity measures after weight loss were calculated. Absolute values and changes in adiposity measures were evaluated as a proxy for achieving an absolute 10‐year ORC risk similar to that of people without obesity, and optimal cut‐offs were identified based on the balance between true positive and true negative rates for achieving the treatment goal.

**Results:**

Absolute values of BMI and WHtR post‐weight change were more strongly associated with 10‐year ORC risk than relative changes; WHtR was the best proxy for a low absolute risk for type 2 diabetes, hypertension, and atherosclerotic cardiovascular disease, and BMI for hip/knee osteoarthritis. Based on the balance between true negative and true positive rates of multiple cut‐offs, a BMI ≤ 27 kg/m^2^ and WHtR ≤ 0.53 are proposed as potential treatment targets for obesity.

**Conclusions:**

These findings suggest that treatment goals for obesity management may be considered on post–weight‐change absolute adiposity measures, rather than relative changes. Both weight and WHtR may be considered when defining treatment targets for ORC risk reduction.

## 1 Introduction

More than 2.5 billion adults are living with overweight and over 890 million are living with obesity worldwide as of 2025 [1, 2, 3, 4, 5, 6, 7]. Obesity‐related complications (ORCs), such as cardiovascular disease, type 2 diabetes, hypertension, and osteorthritis [1, 2, 3, 4, 5, 6], are associated with a substantial health burden for the individual, and an economic burden for individuals and society [1, 3, 8, 9]. Weight loss in individuals with overweight or obesity is associated with a reduced risk of developing ORCs [10, 11, 12, 13], favorable impacts on established ORCs, and improved quality of life and physical function [4, 5, 11, 14, 15]. While increased obesity rates are reported in developed countries, similar trends are also apparent in developing countries, particularly in urban areas [16].

The management of chronic diseases such as type 2 diabetes, hypertension, and dyslipidemia routinely involves the use of treatment targets that help healthcare professionals and patients make decisions regarding initiation, intensification, and stopping of treatment, based on randomized clinical trial data, meta‐analyses, and evidence‐based standards of medical care [17, 18, 19]. These targets are also used by payers to determine the cost‐effectiveness of interventions and by healthcare systems to assess the quality of care delivered.

For chronic metabolic disorders, treatment targets are based on absolute values of the biomarkers used for disease diagnosis (e.g., glycated hemoglobin, blood pressure, or low‐density lipoprotein levels) and are driven by prevention of complications associated with the disease (e.g., cardiovascular disease or retinopathy and other diabetes‐related complications), providing a clear link between the achievement of targets and health benefits [17, 18, 19].

In contrast, in obesity management, there are no widely accepted treatment targets, leading to inconsistent treatment strategies and clinical inertia [20]. Current guidelines typically target percentage weight losses as a goal, ranging from 5% to > 20% depending on the starting weight and goals of therapy [4, 5, 14]. Regulatory guidance for the clinical evaluation of obesity medications indicates ≥ 5% weight loss as a primary endpoint for clinical trials [21, 22], although most patients and healthcare professionals set higher targets [23]. However, compensatory biological and physiologic mechanisms often cause weight regain after weight loss [24]. With the expansion of effective treatment options and increasing numbers of patients receiving treatment for obesity, there is a need for an evidence‐based treatment target to guide healthcare professionals, patients, and payers. This target should be based on a parameter that is routinely measured easily and accurately in the clinic.

This was an observational, population‐based cohort study using data from the UK Clinical Practice Research Datalink (CPRD) Aurum to identify an adiposity measure that could indicate whether obesity management reduces the absolute 10‐year risk of ORCs to a similar level in people without obesity (target risk). Four ORCs, representative of those with a high prevalence and burden among people living with obesity, and encompassing a range of pathophysiologies, were selected for evaluation: type 2 diabetes, hypertension, hip/knee osteoarthritis, and atherosclerotic cardiovascular disease (ASCVD).

## 2 Materials and Methods

### 2.1 Study Design and Patient Population

The study comprised four steps:

1. To define risk functions for estimation of the absolute 10‐year risk of the four selected ORCs in people living with obesity, the association between baseline body mass index (BMI) and waist–height ratio (WHtR), as a surrogate for visceral adiposity, and absolute 10‐year ORC risk (baseline cohort) was examined
2. The association between changes in adiposity measures after weight loss (including BMI and WHtR) and the absolute 10‐year risk of developing the four selected ORCs in people living with obesity (weight‐change cohort) was examined
3. The absolute 10‐year risk of the four selected ORCs for people without obesity, to set the treatment goal (low risk of the ORC) (reference cohort), was determined
4. The performance of absolute values and relative changes in adiposity measures as proxies for achieving the treatment goal defined in step 3 and identified the optimal cut‐offs in the adiposity measures, based on the balance between true positive rates (TPRs) and true negative rates (TNRs), was examined.

### 2.2 Data Source

Data were extracted from the UK CPRD Aurum database, Version 12/2023. CPRD Aurum contains data collected routinely from primary care practices, including diagnoses, symptoms, prescriptions, referrals, tests, and patient electronic healthcare records [25, 26]. As of December 2023, the database included 46,599,092 patients from general practice clinics, including 16,011,762 current patients (23.9% of the UK population). The CPRD Aurum data were merged with patient‐level socioeconomic status and Hospital Episode Statistics data (set 22) to capture any diagnoses from hospital stays. Patient consent was not required as CPRD Aurum data sent by general practice clinics are anonymized. A summary of the study protocol, approved by the CPRD Central Advisory Committee, is available online (Study reference ID: 23_002918: https://www.cprd.com/approved‐studies/treat‐target‐approach‐obesity‐management‐exploring‐potential‐treatment‐targets) [27].

### 2.3 Cohorts

All three cohorts included individuals aged 18–60 years inclusive (individuals older than 60 years were excluded to allow estimation of absolute 10‐year risk without modeling death as a competing outcome) (Supporting Information S1: Figure S1). The index date (baseline and reference cohorts) (the end of the baseline period and start of the follow‐up period) or study start date (weight‐change cohort) was the earliest date between 1 January 2010 and 31 December 2014, when all inclusion criteria were fulfilled. For both the baseline and weight‐change cohorts, individuals with Systematized Nomenclature of Medicine Clinical Terms (SNOMED CT) codes related to cancer, limb amputation, pregnancy, or unintentional weight loss (codes provided in Supporting Information S1: Table S1) during the baseline period were excluded. For all cohorts, individuals were followed up from the index date until diagnosis of their first ORC of interest, or until the end of their record in the database, whichever occurred first.

#### 2.3.1 Baseline Cohort

The baseline cohort included individuals with BMI ≥ 25 kg/m^2^ (with overweight or obesity) at the index date and ≥ 1 BMI and ≥ 1 WHtR measurement during the baseline year (if more than one measurement during the baseline year, the mean was used).

#### 2.3.2 Weight‐Change Cohort

The weight‐change cohort involved a baseline period of 3 years and included individuals with BMI ≥ 25 kg/m^2^ (with overweight or obesity) during baseline year 1 and ≥ 1 BMI and ≥ 1 WHtR measurement during baseline years 1 and 3. Relative changes in anthropometric measures during the baseline period were calculated by comparing mean measurements in year 3 with year 1.

#### 2.3.3 Reference Cohort

For the reference cohort, individuals without obesity (mean BMI 18.5 to < 30 kg/m^2^) in the year prior to the index date were included based on attainability of results. The BMI cut‐offs selected were based on an acceptable low disease risk. No further exclusion criteria were applied. To keep this cohort as similar to the other two cohorts as possible, a waist circumference measurement in the year prior to the index date was required.

### 2.4 Study Variables

Diagnoses of ORCs during follow‐up marked the events in the models. ORCs were identified based on SNOMED CT diagnosis codes in the CPRD Aurum data or International Classification of Diseases, 10th Revision code in the Hospital Episode Statistics data (Supporting Information S1: Table S2). ASCVD was defined as a composite of ischemic heart disease, angina, myocardial infarction, stroke, transient ischemic attack, peripheral artery disease, and cardiac‐atherosclerotic disease. For all cohorts, data included age, sex, race, anthropometric measures (weight, BMI, waist circumference, and WHtR [using patients' most recently reported height]), smoking status, and diagnoses of selected comorbidities (type 2 diabetes, hypertension, dyslipidemia, hip/knee osteoarthritis, and ASCVD). Deprivation measures were used to define socioeconomic status, based on patients' residential postcodes (Supporting Information S1).

Only individuals with a complete data set were included in the full cohort analyses (race, socioeconomic, and smoking status were included in sensitivity analyses in those with data for all three variables).

### 2.5 Statistical Methods

Cox proportional hazards regression analyses were used to assess the association between baseline BMI and WHtR and the absolute 10‐year risk of developing ORCs, with a separate model for each ORC. Individuals with a prior diagnosis of an ORC at baseline were excluded from analyses estimating the risk for that ORC and covariates included were BMI, WHtR, age, sex, and baseline type 2 diabetes, hypertension and dyslipidemia status. To assess the strength of association between BMI and WHtR and ORC risk, BMI and WHtR were standardized so associations per 1 standard deviation (SD) could be compared. Illustrative 2D contour plots were constructed to visualize the relationship between baseline BMI and WHtR and ORC risk (Supporting Information S1).

Cox proportional hazards models were also used to assess the association between BMI and WHtR changes and absolute 10‐year ORC risk, with changes in BMI and WHtR as additional covariates. Illustrative 2D contour plots were constructed as described above. In addition, sensitivity analyses were conducted with smoking status, socioeconomic status, and race as additional covariates, in the subset of individuals (approximately 70%) for whom race and socioeconomic status data were available.

Age‐ and sex‐specific low 10‐year risk of the four ORCs was defined as the observed 10‐year incidence in the reference cohort.

### 2.6 Exploration of Potential Treatment Targets

Area under the receiver operating curve (AUC) analyses were used to compare the performance of different absolute or relative anthropometric adiposity measures as proxies to indicate whether the target ORC risk status (low risk) was reached after weight change. For each ORC, individuals in the weight‐change cohort were categorized as either at or below the target reference risk, or above the low reference risk, after weight change. The ORC models derived from the baseline cohort were used to estimate the ORC risk after weight change.

TPRs and TNRs were calculated for each possible target value of adiposity measures: absolute measures included BMI, WHtR, waist circumference, and weight after weight change; relative change measures included percentage change in weight (BMI) and waist circumference over 3 years. The TNR represents the proportion of patients in the cohort who did not reach the target value for the adiposity measure and who had a higher ORC risk than the target risk (low reference risk) after weight change. The TPR represents the proportion of all patients who reached the target value and had an ORC risk equal to or below the target risk (low reference risk) after weight change.

Receiver operating characteristic curves per adiposity measure were constructed for each ORC, using TPRs and TNRs, and AUCs were calculated for each curve. Potential BMI and WHtR treatment targets were investigated by comparing TPRs with TNRs for each ORC, with the objective of identifying a target that maximizes both. Combining BMI and WHtR measures into a single two‐component treatment target was also explored using a similar methodology. Further information is provided in the Supporting Information S1.

## 3 Results

### 3.1 Association Between Baseline BMI, WHtR, and Absolute 10‐Year ORC Risk

The baseline cohort included 499,813 individuals (Table 1, Supporting Information S1: Figure S2). Median age at baseline was 48 years, and there were 12.9, 33.7, 11.0, and 8.6 incident diagnoses per 1000 patient‐years of type 2 diabetes, hypertension, hip/knee osteoarthritis, and ASCVD, respectively, during a median follow‐up duration of 9.9 years (Table 1).

**TABLE 1:** Characteristics of the baseline cohort.

	Total	Overweight	Obesity I	Obesity II	Obesity III
*N*	499,813	257,211	145,467	60,930	36,205
Women	233,454 (46.7%)	107,880 (41.9%)	67,633 (46.5%)	34,655 (56.9%)	23,286 (64.3%)
Men	266,359 (53.3%)	149,331 (58.1%)	77,834 (53.5%)	26,275 (43.1%)	12,919 (35.7%)
Baseline characteristics, median (IQR)					
Age, years	48 (41–54)	48 (41–54)	49 (42–55)	49 (42–55)	48 (41–54)
Weight, kg	87 (77–99)	79 (72–87)	92 (84–101)	104 (95–114)	122 (111–135)
BMI, kg/m^2^	29.8 (27.2–33.7)	27.3 (26.1–28.5)	32.0 (30.9–33.3)	36.9 (35.9–38.3)	43.4 (41.4–46.6)
Waist circumference, cm	99 (91–108)	93 (86–99)	104 (97–110)	113 (106–120)	125 (116–135)
WHtR	0.58 (0.54–0.64)	0.54 (0.51–0.57)	0.61 (0.58–0.64)	0.67 (0.64–0.71)	0.75 (0.71–0.80)
Follow‐up, years	9.9 (6.4–11.9)	9.7 (6.1–11.7)	10.1 (6.7–12.0)	10.2 (6.8–12.3)	10.2 (6.8–12.3)
Smoking status					
Current	94,633 (18.9%)	50,703 (19.7%)	27,020 (18.6%)	10,832 (17.8%)	6078 (16.8%)
Ex	143,552 (28.7%)	67,966 (26.4%)	44,001 (30.2%)	19,449 (31.9%)	12,136 (33.5%)
Never	197,920 (39.6%)	101,776 (39.6%)	57,265 (39.4%)	24,345 (40.0%)	14,534 (40.1%)
Unknown	63,708 (12.7%)	36,766 (14.3%)	17,181 (11.8%)	6304 (10.3%)	3457 (9.5%)
ORCs at start of follow‐up					
Type 2 diabetes	76,433 (15.3%)	22,255 (8.7%)	25,511 (17.5%)	16,153 (26.5%)	12,514 (34.6%)
Hypertension	131,320 (26.3%)	45,916 (17.9%)	43,938 (30.2%)	24,371 (40.0%)	17,095 (47.2%)
Dyslipidemia	81,818 (16.4%)	30,322 (11.8%)	27,906 (19.2%)	14,195 (23.3%)	9395 (25.9%)
Hip/knee osteoarthritis	22,119 (4.4%)	7498 (2.9%)	7232 (5.0%)	4309 (7.1%)	3080 (8.5%)
ASCVD	26,652 (5.3%)	9928 (3.9%)	9155 (6.3%)	4587 (7.5%)	2982 (8.2%)
Ethnicity					
White	376,790 (75.4%)	188,352 (73.2%)	110,117 (75.7%)	48,239 (79.2%)	30,082 (83.1%)
Asian	46,841 (9.4%)	28,648 (11.1%)	12,722 (8.7%)	3917 (6.4%)	1554 (4.3%)
Black	32,429 (6.5%)	15,757 (6.1%)	10,285 (7.1%)	4142 (6.8%)	2245 (6.2%)
Unknown	43,753 (8.8%)	24,454 (9.5%)	12,343 (8.5%)	4632 (7.6%)	2324 (6.4%)
Patient residence area socioeconomic status					
IMD 1	51,966 (10.4%)	29,189 (11.3%)	14,492 (10.0%)	5453 (8.9%)	2832 (7.8%)
IMD 2	57,156 (11.4%)	29,815 (11.6%)	16,732 (11.5%)	6812 (11.2%)	3797 (10.5%)
IMD 3	59,312 (11.9%)	29,601 (11.5%)	17,711 (12.2%)	7518 (12.3%)	4482 (12.4%)
IMD 4	73,305 (14.7%)	34,453 (13.4%)	22,283 (15.3%)	10,123 (16.6%)	6446 (17.8%)
IMD 5	79,030 (15.8%)	34,603 (13.5%)	24,301 (16.7%)	12,220 (20.1%)	7906 (21.8%)
IMD unknown	179,044 (35.8%)	99,550 (38.7%)	49,948 (34.3%)	18,804 (30.9%)	10,742 (29.7%)
Diagnoses during follow‐up per 1000 patient‐years					
Type 2 diabetes	12.9	7.3	15.8	25.8	33.6
Hypertension	33.7	26.6	39.7	48.7	56.9
Hip/knee osteoarthritis	11.0	7.9	12.1	16.6	20.3
ASCVD	8.6	7.2	9.6	10.9	11.6

*Note:* Overweight, BMI 25 to < 30 kg/m^2^; Obesity I, BMI 30 to < 35 kg/m^2^; Obesity II, 35 to < 40 kg/m^2^; Obesity III, ≥ 40 kg/m^2^.

Abbreviations: ASCVD, atherosclerotic cardiovascular disease; BMI, body mass index; IMD, Index of Multiple Deprivation; IQR, interquartile range; ORC, obesity‐related complication; WHtR, waist–height ratio.

The association between 1 SD higher BMI and WHtR at baseline and absolute risk was different for each of the four ORCs (Supporting Information S1: Figure S3). A baseline WHtR of 1 SD higher was more strongly associated with the risk of type 2 diabetes, hypertension, and ASCVD than a baseline BMI of 1 SD higher. In contrast, a baseline BMI of 1 SD higher was more strongly associated with risk of hip/knee osteoarthritis than a baseline WHtR of 1 SD higher. The associations between other covariates and risk of ORCs are also shown in Supporting Information S1: Figure S3. Men had a higher risk of type 2 diabetes, hypertension, and ASCVD than women, and a lower risk of hip/knee osteoarthritis. The risk of all four ORCs increased with age. The presence of hypertension and dyslipidemia at baseline increased the risk of type 2 diabetes and ASCVD; the presence of type 2 diabetes at baseline increased the risk of hypertension and ASCVD.

**FIGURE 1:**
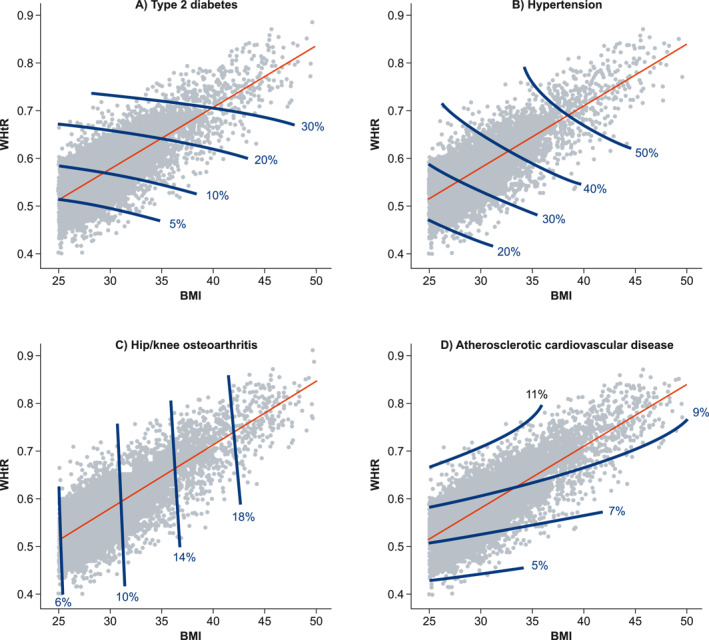
Illustrative contour plots of the relationship between baseline WHtR, BMI, and absolute risk of ORCs for 50‐year‐old men for (A) type 2 diabetes, (B) hypertension, (C) hip/knee osteoarthritis, and (D) atherosclerotic cardiovascular disease. The gray points are combinations of BMI and WHtR for 50‐year‐old men in the study cohort; the red line represents the linear fit across the points; and the contour lines visualize the absolute 10‐year ORC risk for the BMI and WHtR combinations. BMI, body mass index; ORC, obesity‐related complication; WHtR, waist–height ratio.

Example contour plots illustrating the contributions of BMI and WHtR to risk for the four ORCs are presented in Figure 1. Both BMI and WHtR contributed to the risks of type 2 diabetes and hypertension, as shown by the diagonal contour lines in these plots: patients with the same BMI may have different risks depending on their WHtR (and vice versa). Only BMI contributed to hip/knee osteoarthritis risk, and only WHtR contributed to ASCVD risk.

### 3.2 Association Between Changes in BMI and WHtR and Absolute 10‐Year ORC Risk

The weight‐change cohort used for analyzing the association between weight loss and absolute ORC risk included 45,899 individuals (Table 2, Supporting Information S1: Figure S2). Median age at the index date was 51 years, and relative to the baseline cohort, individuals in this subset had a higher prevalence of ORCs at the start of follow‐up. During median follow‐up of 8.1 years, there were 17.5, 45.5, 14.7, and 12.5 incident diagnoses per 1000 patient‐years of type 2 diabetes, hypertension, hip/knee osteoarthritis, and ASCVD, respectively (Table 2). Characteristics for the subset of individuals with complete socioeconomic status, and race data available (*N* = 32,026) are presented in Supporting Information S1: Table S3.

**TABLE 2:** Characteristics of the weight‐change cohort.

	Total	Overweight	Obesity I	Obesity II	Obesity III
*N*	45,899	18,808	15,029	7475	4587
Women	20,563 (44.8%)	7459 (39.7%)	6482 (43.1%)	3873 (51.8%)	2749 (59.9%)
Men	25,336 (55.2%)	11,349 (60.3%)	8547 (56.9%)	3602 (48.2%)	1838 (40.1%)
Baseline characteristics, median (IQR)					
Age at index date, years	51 (44–56)	51 (44–56)	51 (45–56)	51 (44–56)	50 (43–55)
BMI year 1, kg/m^2^	31.1 (28.1–35.3)	27.6 (26.4–28.8)	32.1 (31.0–33.4)	37.0 (35.9–38.3)	43.3 (41.3–46.2)
BMI year 3, kg/m^2^	31.2 (28.1–35.4)	27.7 (26.3–29.2)	32.2 (30.7–33.8)	36.9 (35.3–38.7)	43.0 (40.5–46.2)
BMI change, %	0.1 (−3.3–3.6)	0.6 (−2.5–4.0)	0.0 (−3.3–3.6)	−0.2 (−3.9–3.1)	−1.0 (−5.3–2.9)
WHtR year 1	0.61 (0.56–0.67)	0.56 (0.53–0.59)	0.62 (0.59–0.65)	0.68 (0.65–0.72)	0.76 (0.72–0.80)
WHtR year 3	0.61 (0.56–0.67)	0.56 (0.53–0.59)	0.62 (0.59–0.66)	0.69 (0.65–0.72)	0.76 (0.71–0.80)
WHtR change, %	0.3 (−3.1–4.4)	0.5 (−2.7–4.7)	0.3 (−3.1–4.3)	0.1 (−3.5–4.0)	0.0 (−4.0–3.7)
Follow‐up, years	8.1 (6.1–9.8)	8.0 (6.1–9.7)	8.2 (6.2–9.9)	8.3 (6.2–9.9)	8.1 (6.0–9.9)
Smoking status					
Current	6618 (14.4%)	2838 (15.1%)	2175 (14.5%)	994 (13.3%)	611 (13.3%)
Ex	18,296 (39.9%)	7077 (37.6%)	6113 (40.7%)	3154 (42.2%)	1952 (42.6%)
Never	18,750 (40.9%)	7944 (42.2%)	6039 (40.2%)	2980 (39.9%)	1787 (39.0%)
Unknown	2235 (4.9%)	949 (5.0%)	702 (4.7%)	347 (4.6%)	237 (5.2%)
Weight‐loss interventions during baseline period					
Weight‐loss drugs	2731 (6.0%)	235 (1.2%)	811 (5.4%)	841 (11.3%)	844 (18.4%)
Bariatric surgery	173 (0.4%)	4 (0.0%)	13 (0.1%)	34 (0.5%)	122 (2.7%)
Ethnicity					
White	34,046 (74.2%)	12,990 (69.1%)	11,262 (74.9%)	5944 (79.5%)	3850 (83.9%)
Asian	5696 (12.4%)	3096 (16.5%)	1738 (11.6%)	638 (8.5%)	224 (4.9%)
Black	2468 (5.4%)	1034 (5.5%)	835 (5.6%)	375 (5.0%)	224 (4.9%)
Unknown	3689 (8.0%)	1688 (9.0%)	1194 (7.9%)	518 (6.9%)	289 (6.3%)
Patient residence area socioeconomic status					
IMD 1	5453 (11.9%)	2406 (12.8%)	1822 (12.1%)	816 (10.9%)	409 (8.9%)
IMD 2	5779 (12.6%)	2449 (13.0%)	1890 (12.6%)	928 (12.4%)	512 (11.2%)
IMD 3	6187 (13.5%)	2534 (13.5%)	2013 (13.4%)	1014 (13.6%)	626 (13.6%)
IMD 4	8220 (17.9%)	3340 (17.8%)	2606 (17.3%)	1368 (18.3%)	906 (19.8%)
IMD 5	9279 (20.2%)	3601 (19.1%)	2988 (19.9%)	1659 (22.2%)	1031 (22.5%)
IMD unknown	10,981 (23.9%)	4478 (23.8%)	3710 (24.7%)	1690 (22.6%)	1103 (24.0%)
ORCs at start of follow‐up					
Type 2 diabetes	21,494 (46.8%)	6642 (35.3%)	7323 (48.7%)	4502 (60.2%)	3027 (66.0%)
Hypertension	26,433 (57.6%)	9153 (48.7%)	9037 (60.1%)	5007 (67.0%)	3236 (70.5%)
Dyslipidemia	21,556 (47.0%)	7810 (41.5%)	7460 (49.6%)	3936 (52.7%)	2350 (51.2%)
Hip/knee osteoarthritis	4272 (9.3%)	1134 (6.0%)	1470 (9.8%)	985 (13.2%)	683 (14.9%)
ASCVD	6079 (13.2%)	2317 (12.3%)	2062 (13.7%)	1057 (14.1%)	643 (14.0%)
Diagnoses during follow‐up per 1000 patient‐years					
Type 2 diabetes	17.5	11.3	19.9	29.5	34.9
Hypertension	45.5	37.7	49.2	57.7	67.1
Hip/knee osteoarthritis	14.7	10.6	15.9	18.5	23.4
ASCVD	12.5	11.1	13.3	13.8	13.7

*Note:* Overweight, BMI 25 to < 30 kg/m^2^; Obesity I, BMI 30 to < 35 kg/m^2^; Obesity II, 35 to < 40 kg/m^2^; Obesity III, ≥ 40 kg/m^2^.

Abbreviations: ASCVD, atherosclerotic cardiovascular disease; BMI, body mass index; IMD, Index of Multiple Deprivation; IQR, interquartile range; ORC, obesity‐related complication; WHtR, waist–height ratio.

There was a positive correlation between changes in BMI and WHtR. However, there was heterogeneity between individuals, with a range of WHtR changes for any given BMI change (Supporting Information S1: Figure S4). Analyzing the 10‐year risk of developing ORCs showed that, whereas both BMI and WHtR reductions were associated with reductions in type 2 diabetes and hypertension risk, only BMI reductions were associated with reduced hip/knee osteoarthritis risk and only WHtR reductions were associated with reduced ASCVD risk (Supporting Information S1: Figure S5). Findings of the additional Cox proportional hazard model analyses incorporating smoking status, socioeconomic status and race, were consistent with primary analyses in the weight‐change cohort (Supporting Information S1: Figure S6). Notably, the risk of ORCs tended to be higher in non‐White populations, and particularly in the Asian population.

### 3.3 Defining Absolute 10‐Year ORC Risk in People Without Obesity

The reference cohort used to derive low absolute 10‐year ORC risk (target/reference risk) status included 318,178 individuals (Supporting Information S1: Table S4). At the index date, median age was 49 years, BMI was 25.6 kg/m^2^, and WHtR was 0.52. The target risk is defined as the sex‐specific 10‐year ORC incidence according to age, as shown in Supporting Information S1: Figure S7.

### 3.4 Exploration of Potential Treatment Targets

**FIGURE 2:**
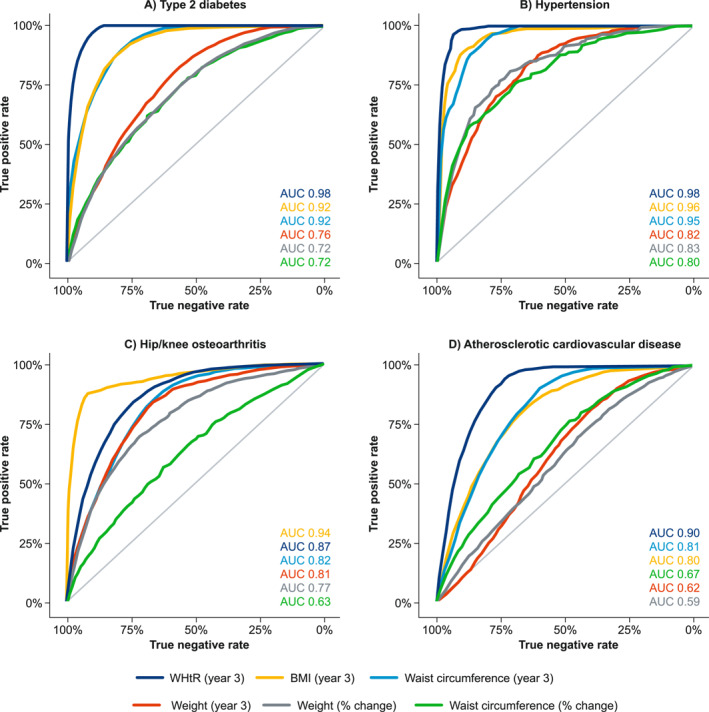
Receiver operating characteristic curves assessing the potential for absolute and relative measures to indicate whether a low risk of ORCs was reached in the weight‐change cohort (*N* = 45,899). AUC for absolute measurement at year 3 (BMI, WHtR, weight or waist circumference), and percentage change in weight or waist circumference from baseline to year 3 were plotted for each ORC. A low reference risk was defined as the age‐ and sex‐stratified 10‐year ORC incidence in the reference cohort (see Supporting Information S1: Figure S7). AUC, area under the receiver operating curve; BMI, body mass index; ORC, obesity‐related complication; WHtR, waist–height ratio.

AUC analyses in the weight‐change cohort (*N* = 45,899), assessing the performance of different adiposity measures for indicating achievement of a low ORC risk status, are presented in Figure 2. Performance was better (higher AUC) for absolute measures of BMI and WHtR after weight change than for percentage changes in weight and waist circumference for all four ORCs. For type 2 diabetes and hypertension, AUC was ≥ 0.90 for both absolute post–weight‐change BMI and WHtR. For hip/knee osteoarthritis, AUC was higher for absolute BMI (0.94) than WHtR (0.87) after weight change. For ASCVD, the converse was true, with higher AUC for absolute WHtR (0.90) than BMI (0.80) after weight change.

The performance of potential different BMI and WHtR targets for indicating the achievement of a low absolute 10‐year risk of developing ORCs is shown in Supporting Information S1: Figure S8. Although different targets could be chosen for different ORCs, the aim was to identify one target that performed reasonably well across all four. For the proposed targets of a BMI ≤ 27 mg/kg^2^ or WHtR ≤ 0.53, the TNR was above 80% across all four ORCs, whereas TPR was more variable (Supporting Information S1: Table S5). Specifically, the BMI target had low TPR for ASCVD and the WHtR target had low TPR for osteoarthritis. Using a combination of BMI and WHtR did not improve the target performance (Supporting Information S1: Table S6).

## 4 Discussion

This study showed that absolute values of BMI and WHtR post‐weight change were more closely associated with the absolute 10‐year risk of type 2 diabetes, hypertension, hip/knee osteoarthritis, and ASCVD than with percentage changes in weight or waist circumference. Post–weight‐change WHtR was more strongly associated with the 10‐year risk of type 2 diabetes, hypertension, and ASCVD than was BMI; BMI was more strongly associated with the risk of hip/knee osteoarthritis. Based on these results, we propose a BMI ≤ 27 kg/m^2^ or a WHtR ≤ 0.53 as a treatment target for obesity management. These findings highlight the importance of routine WHtR measurement in clinical practice, and that BMI should not be measured in isolation.

A treatment target was identified in this study by linking a specific biomarker value used for disease diagnosis with the long‐term risk of disease complications. As with other chronic metabolic disorders, the results showed that post‐change absolute values of the potential treatment target best reflect the risk of disease complications. However, unlike type 2 diabetes and hypertension, for which treatment targets are based on randomized controlled trials measuring the risk of diabetes complications [28, 29] or CVD [30], this study used real‐world evidence. Hence, these potential treatment targets need to be tested in randomized controlled trials. Also, the identified treatment targets are based on prevention of ORCs; validation assessing their impact on established ORCs is needed.

The identified potential treatment targets of BMI ≤ 27 kg/m^2^ and WHtR ≤ 0.53 were chosen based on the balance of TNR and TPR, to maximize the proportion of patients receiving appropriate treatment intensification while minimizing over‐treatment in those who achieved the low‐risk category. Hence, these findings are sensitive to both TNR and TPR. The study should be repeated in cohorts for which effective obesity treatment options are offered, where there would be a greater proportion of patients achieving treatment targets.

A low‐risk status for future ORCs may not be feasible for all people living with obesity, even if the treatment target is achieved, as residual risk related to unmeasured factors other than obesity (such as genetics or sedentary behavior) might contribute. Obesity management should be seen as integral to lowering the risk of future ORCs but is not the sole intervention needed; other risk factors still needs to be addressed. It was recently shown that a variety of risk factors contribute to the development of osteoarthritis, despite obesity being a prominent risk factor in some phenotypes [31]. Another contributor to residual risk could be the duration of obesity, which is difficult to identify in real‐world databases or clinical trial populations. Understanding how obesity duration might modify treatment outcomes in obesity management requires investigation.

A low‐risk status or the treatment target might not be achievable for every person living with obesity and it is important that healthcare professionals and patients do not feel they have failed if targets are not met. Healthcare professionals and patients should be mindful that even small weight loss is associated with benefit, even if the final BMI or WHtR remains above the identified target.

In type 2 diabetes, guidelines give an average treatment target but suggest that it should be individually adjusted [17]. The need for a personalized approach is supported by our findings that associations between post‐treatment adiposity measures and ORC risk vary. For example, WHtR was more strongly linked to ASCVD risk, whereas BMI was more strongly linked to osteoarthritis risk. However, while BMI is an established measurement, waist circumference (and therefore WHtR) is measured less frequently in clinical practice, which may lead to failure in recognizing some patients with high cardiometabolic risk. It will be imperative to include both BMI and WHtR for obesity, with individualized targets for clinicians to use as deemed relevant to their patients, e.g., where CV risk is the primary concern, the focus should be on the patient reducing their waist circumference, perhaps to reach a WHtR at or below the 0.53 suggested. However, where there is a family history of osteoarthritis, a BMI target is likely to be more relevant.

In clinical practice and regulatory processes, the efficacy of weight‐loss interventions is largely based on percentage weight loss, but our findings do not support this as a treatment target. The correlation between visceral adiposity and risk of ORCs is well acknowledged [32, 33] and is reflected in updated National Institute for Health and Care Excellence guidelines recommending that people with WHtR ≥ 0.5 seek advice and clinical assessment from a healthcare professional [34]. The European Association for the Study of Obesity also considers WHtR > 0.5 as a new framework for the diagnosis, staging, and management of obesity [35, 36]. Other guidelines acknowledge the importance of body composition and waist circumference as part of patient evaluation [4, 5] but do not propose targets. Previous studies have demonstrated a strong link between WHtR and CVD and found that reducing WHtR can modify the association between body weight and ORCs [37, 38]. Our findings suggest that absolute measures of visceral adiposity should be integrated in treatment goals for obesity management. “Distance to target” should be an important consideration when choosing an obesity intervention, e.g., when choosing an obesity intervention/treatment for people with a higher BMI or WHtR, interventions or treatments with greater efficacy may be needed.

The proposed treatment targets for obesity management are derived by linking the target to ORCs, in line with the approach used in other chronic diseases. This was based on four ORCs, and it is unknown whether other ORCs would have produced the same results. However, the ORCs used in this study are highly prevalent and represent a major burden. The CPRD Aurum was used as it provided a large sample of a well‐characterized population, with long‐term follow‐up. However, the findings need to be validated in other healthcare systems and populations with multiple ethnicities. The inclusion of repeated waist measurements might have introduced a selection bias, given that waist circumference is less frequently measured than BMI in real practice and the rationale for repeated measurements in a subset of patients is unknown. The lack of hip measurements prevented us from including the waist–hip ratio in this analysis. Although individuals with cancer and unintentional weight loss were excluded from the weight‐change cohort, residual confounding from unintentional weight loss from other chronic diseases is still possible. This may include endocrine diseases and eating‐behavior disorders, which can cause individuals to lose weight, as well as individuals taking certain mediation (e.g., for attention deficit hyperactivity disorder), which may reduce appetite through the presence of amphetamine‐related components. In addition, most of the weight change in this study was not driven by bariatric surgery or pharmacotherapy (based on patient records from 2010 to 2014). As such, further validation studies need to re‐examine the targets within the context of specific obesity interventions, especially if these might impact ORCs beyond weight loss. Moreover, it is known that individuals aged over 60 years may experience unintentional weight loss and therefore, these results cannot be extrapolated to those older than 60 years. Ethnicity‐specific thresholds were not accounted for specifically in this analysis. It is known that South Asian, Chinese, and Black individuals can develop diabetes at lower BMI thresholds than White individuals [39]. It should be noted that while AUC values were high for some outcomes (≥ 0.90), receiver operating characteristic analyses do not account for calibration, and the clinical utility of these targets in real‐world practice remains uncertain without further external validation. While adjustments were made for age, sex, and baseline metabolic conditions, other confounders such as dietary patterns, physical activity, and genetic predisposition were not available in primary care records and could not be accounted for. Several traditional CV risk factors were not included in the ASCVD model, which is the subject of further research. Finally, this study was observational and cannot therefore be used to establish causality.

This analysis of real‐world clinical practice data suggests that treatment goals for obesity management should be based on absolute anthropometric adiposity measures rather than relative changes when aiming for a low‐risk status for the development of ORCs. It is important to consider both BMI and WHtR when defining potential treatment targets. We have proposed treatment targets for hypothesis gathering purposes at this stage that will require validation in randomized controlled trials across different populations and treatment approaches, but which set the scene for a treat‐to‐target approach in obesity management to align with the management of other chronic metabolic disorders. Incorporating absolute anthropometric measures into obesity management aligns with strategies in other chronic conditions.

## Author Contributions

A.A.T., C.S.M., L.B., S.C., and V.S. designed the study, and V.S. conducted the data analysis. All authors contributed to the data interpretation and manuscript writing (assisted by a medical writer paid for by the funder), approved the final version of the manuscript, and vouch for data accuracy and fidelity to the protocol.

## Conflicts of Interest

L.B. received consulting fees from Novo Nordisk, Eli Lilly, Bruno Farmaceutici, Boehringer Ingelheim, and Pfizer and received honoraria from Rhythms Pharmaceuticals and Pronokal, and did not receive any consulting fees related to this publication. V.S., M.O., S.C., R.R., R.B., and C.S.M. are employees and shareholders of Novo Nordisk. A.A.T. was an employee and shareholder of Novo Nordisk at the time of writing the manuscript. A.A.T. is currently an employee of Amgen Research Copenhagen (ARC). ARC had no role in this project and manuscript.

## Supporting information


Supporting Information S1

## Data Availability

This study is based on data from the UK Clinical Practice Research Datalink Aurum obtained under license from the UK Medicines and Healthcare products Regulatory Agency. The data are provided by patients and collected by the UK National Health Service as part of their care and support. The interpretation and conclusions contained in this study are those of the authors alone. Electronic health records are classified as “sensitive data” by the UK Data Protection Act; therefore, information governance restrictions prevent data sharing via public deposition. Information about access to CPRD Aurum data is available here: https://www.cprd.com/research‐applications (accessed 13 June 2022).
