# Supplementary material for: The Identification of Potential Treatment Targets to Reduce the Risk of Obesity‐Related Complications: A Step Toward a Treat‐to‐Target Approach in Obesity Management
Source: Obes Sci Pract. 2025 Nov 5;11(6):e70094. doi: 10.1002/osp4.70094 (PMC12589817; doi:10.1002/osp4.70094)
Supplement: Supplementary file 1 — Supporting Information S1 [file OSP4-11-e70094-s001.docx]

Supporting Information

**The identification of potential treatment targets to reduce the risk of obesity-related complications: a step towards a treat-to-target approach in obesity management**

Luca Busetto PhD^1^, Volker Schnecke PhD^2^, Maria Overvad PhD^2^, Silvia Capucci MSc^2^, Ricardo Reynoso MD^3^, Rafael Bravo MD^2^, Abd A. Tahrani PhD^2,4^*, Camilla S. Morgen PhD^2^*

^1^Department of Medicine, University of Padova, Padova, Italy

^2^Novo Nordisk A/S, Søborg, Denmark

^3^Novo Nordisk Health Care, Zürich, Switzerland

^4^Metabolism and Systems Science, University of Birmingham, Birmingham, UK

*Camilla S. Morgen and Abd A. Tahrani should be considered joint senior author.

**Correspondence**
Luca Busetto, Clinica Medica 3, Azienda Ospedale Università di Padova, Via Giustiniani 2, 35128 Padova, Italy

Tel: +39 049 8218250

Email: luca.busetto@unipd.it

Contents

[Patient postcode-linked deprivation measures 3](#_Toc202533198)

[Additional statistical methods 4](#_Toc202533199)

[Table S1. SNOMED CT codes used to identify unintentional weight loss 7](#_Toc202533200)

[Table S2. Diagnosis codes used to identify obesity-related complications 8](#_Toc202533201)

[Table S3. Characteristics of the cohort used for the additional cox proportional hazards model analyses incorporating smoking status, socioeconomic status and race 10](#_Toc202533202)

[Table S4. Characteristics of the reference cohort used to derive the 10-year low obesity-related complication risk 13](#_Toc202533203)

[Table S5. True positive and true negative rates for potential BMI and WHtR targets, alone and combined, as indicators of a low absolute 10-year risk of ORCs after weight loss in the weight-change cohort (*N* = 45,899) 14](#_Toc202533204)

[Table S6. Characteristics of the baseline cohort and a cohort based on individuals with a BMI record only, no waist (or WHrR) measurement, during 2010 and 2014. 15](#_Toc202533205)

[Figure S1. Study designs 16](#_Toc202533206)

[Figure S2. Participant disposition 17](#_Toc202533207)

[Figure S3. Association between baseline characteristics and obesity-related complication hazard ratio in the baseline cohort (*N* = 499,813) 18](#_Toc202533208)

[Figure S4. Changes in BMI and WHtR during follow-up in the cohort used for analyses of the associations between weight loss and obesity-related complication risk (*N* = 45,899) 19](#_Toc202533210)

[Figure S5. Pattern of 10-year risk of obesity-related complications by BMI and WHtR change for women aged 50 years with BMI 33 kg/m^2^ and WHtR 0.61 in the weight-change cohort 20](#_Toc202533211)

[Figure S6. Contribution of the covariates in the additional Cox proportional hazard model for the weight-change cohort, including smoking status, race and socioeconomic status (*N* = 32,026) 21](#_Toc202533212)

[Figure S7. Sex-specific 10-year incidence of obesity-related complications by age for the reference cohort of individuals without obesity (*N* = 318,178) 22](#_Toc202533213)

[Figure S8. Accuracy for BMI and WHtR as treatment targets for achieving a low reference 10-year risk of obesity-related complications in the weight-change cohort (*N* = 45,899) 23](#_Toc202533214)

## Patient postcode-linked deprivation measures

Patient postcode-linked measures are available for patients in English practices that have consented to participate in the linkage scheme. The latest available patient postcode of residence is mapped to a lower layer super output area (LSOA) boundary. The LSOA of residence then allows linkage to the following LSOA-level deprivation measures:

- 2019 English Index of Multiple Deprivation (composite and individual domains)
- Townsend Deprivation Index: calculated using unadjusted 2011 census data
- Carstairs Index: uses 2011 census data

Data are provided as quintiles, deciles or twentiles of the deprivation score to prevent disclosure of patient location. In order to prevent the possibility of deductive disclosure of a patient’s area of residence, researchers are provided with only one of the above linked datasets for any one study. Access is provided by Clinical Practice Research Datalink, subject to approval.

## Additional statistical methods

***Cox proportional hazard models***

The main models used in these studies were based on the baseline cohort, i.e., estimating the 10-year obesity-related complication (ORC) risk based on a single body mass index (BMI) and waist–height ratio (WHtR) data point. All models included BMI, WHtR, and sex. Baseline comorbidities, quadratic terms for the anthropometric measures, and interactions between these variables and sex were only included when they contributed significantly to the model. Age was used as the underlying time variable. The proportional hazards assumption for the variables was assessed by inspecting Schoenfeld residuals and creating Kaplan-Meier curves for the stratified cohort. Only records with complete data were used for modeling, no imputation of any parameter was conducted.

**Parameters included in the baseline ORC models**

| ORC | Parameter | Hazard ratio | 95% CI |
| --- | --- | --- | --- |
| Type 2 diabetes | BMI | 1.038 | 1.034 1.042 |
|  | WHtR (%) | 1.096 | 1.093 1.099 |
|  | Sex (male) | 1.440 | 1.413 1.468 |
|  | Hypertension | 1.506 | 1.475 1.539 |
|  | Dyslipidemia | 1.501 | 1.464 1.539 |
|  | Quadratic term WHtR (%) | 0.999 | 0.999 0.999 |
|  | Interaction BMI : WHtR | 0.998 | 0.998 0.999 |
| Hypertension | BMI | 1.038 | 1.035 1.041 |
|  | WHtR (%) | 1.034 | 1.032 1.036 |
|  | Sex (male) | 1.302 | 1.285 1.320 |
|  | Type 2 diabetes | 2.033 | 1.992 2.075 |
|  | Dyslipidemia | 1.168 | 1.143 1.193 |
|  | Quadratic term BMI (%) | 0.999 | 0.999 1.000 |
|  | Quadratic term WHtR (%) | 0.999 | 0.999 1.000 |
|  | Interaction BMI : WHtR | 1.000 | 0.999 1.000 |
| Hip/knee osteoarthritis | BMI | 1.083 | 1.078 1.087 |
|  | WHtR (%) | 1.002 | 1.000 1.004 |
|  | Sex (male) | 0.684 | 0.671 0.697 |
|  | Type 2 diabetes | 0.799 | 0.778 0.821 |
|  | Dyslipidemia | 1.048 | 1.022 1.074 |
|  | Quadratic term BMI (%) | 0.998 | 0.998 0.998 |
| ASCVD | BMI | 0.986 | 0.983 0.990 |
|  | WHtR (%) | 1.038 | 1.035 1.041 |
|  | Sex (male) | 1.635 | 1.595 1.676 |
|  | Type 2 diabetes | 1.632 | 1.589 1.677 |
|  | Hypertension | 1.493 | 1.457 1.530 |
|  | Dyslipidemia | 1.310 | 1.275 1.345 |
|  | Quadratic term WHtR (%) | 0.999 | 0.999 0.999 |
|  | Interaction BMI : Sex | 0.993 | 0.991 0.996 |

ASCVD, atherosclerotic cardiovascular disease; BMI, body mass index; CI, confidence interval; ORC, obesity-related complication; WHtR, waist–height ratio.

***Contour plot generation and interpretation***


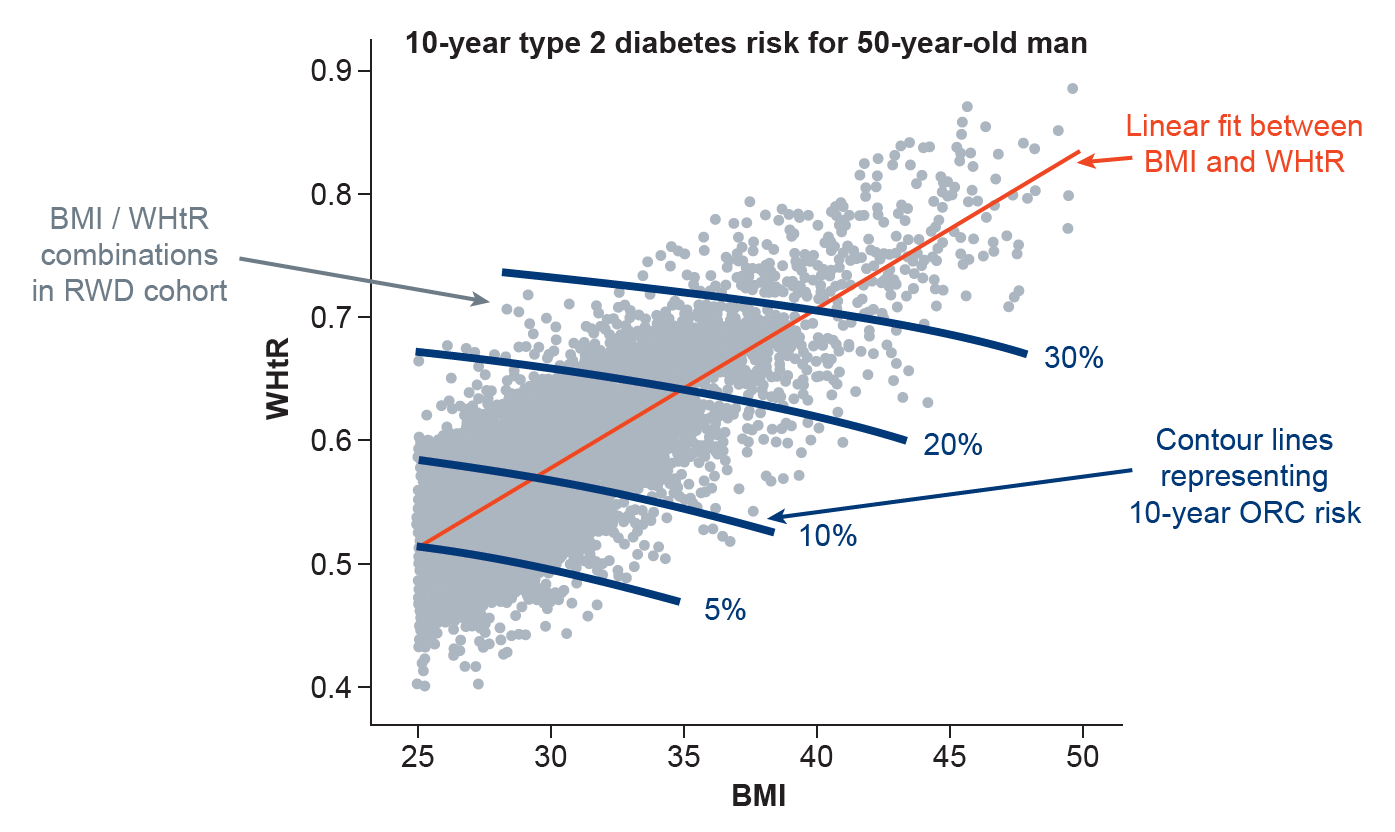

BMI, body mass index; ORC, obesity-related complication; WHtR, waist–height ratio.

The primary covariates in the risk models are BMI and WHtR. To visualize the association between ORC risk and these adiposity measures, 2D contour plots are used. The x-axis represents the BMI range from 25 to 50, and the y-axis represents the WHtR range from 0.4 to 0.9. Each grey dot in the plot represents an individual from the study cohort. Because sex and age are significant covariates that influence absolute ORC risk, it is essential to fix these covariates when displaying the risk in a 2D contour plot. In the above plot, BMI/WHtR combinations for 50-year-old men only are shown. The red line represents a linear fit between BMI and WHtR, indicating the mean WHtR observed in this group of 50-year-old men at a given BMI. To visualize the pattern for the ORC risk across the BMI and WHtR ranges, contour lines are drawn. This involves computing the baseline ORC risk on a grid for all combinations of the BMI and WHtR ranges for the 50-year-old men. Subsequently, the geom_contour() function in the ggplot2 R package is used to generate the 2D contour lines across the full plot area, to visualize the pattern in which the ORC risk increases with BMI and WHtR.

***Generation of ROC curves and TPR/TNR calculation/interpretation***

When comparing various relative and absolute adiposity measures and selecting specific values for these measures, we utilize true positive rates (TPRs) and true negative rates (TNRs), which are commonly visualized through receiver operating characteristic (ROC) curves. To construct ROC curves, such as for BMI, we range from the lowest feasible value (e.g. 23) to the highest feasible value (e.g. 50) and determine the TPR and TNR at each value.


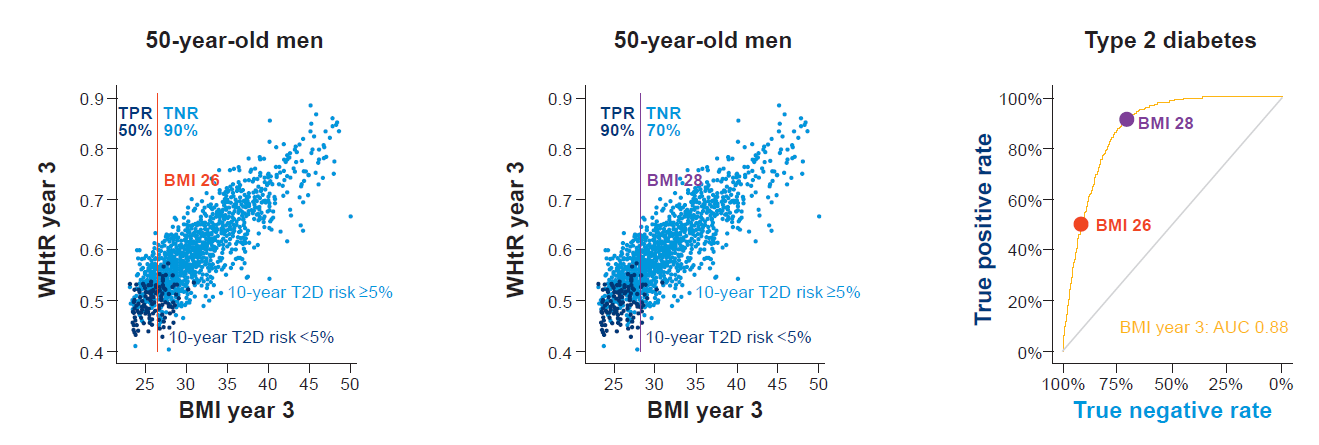


AUC, area under the curve; BMI, body mass index; T2D, type 2 diabetes; TNR, true negative rate; TPR, true positive rate; WHtR, waist–height ratio.

Displaying the combinations of BMI and WHtR in a 2D scatterplot with the x-axis representing BMI, we have a vertical divider that separates all data points into two groups. The dark blue points on the leftmost plot represent individuals with a type 2 diabetes risk below the low reference risk, which is 5% for 50-year-old men, and the light blue points represent individuals with an estimated risk above 5%. At BMI 26 kg/m^2^, 50% of the individuals in the low-risk class are to the left of the divider, representing the true positives, and 90% of the high-risk class are on the right of the divider, representing the true negatives. By iterating this procedure through all possible BMI values and marking the TPR/TNR combination in a 2D plot, with the x-axis in reversed scale representing the TNR and the y-axis representing TPR, we can draw an ROC curve, as shown in the right-hand graph above. The area under the ROC curve provides a measure of the accuracy of using BMI to separate the two classes we are investigating. Using the same example scatterplot and drawing a horizontal divider that we move from the lowest WHtR value of 0.4 to the highest value of 0.9 and marking the TPR/TNR combination for each possible WHtR value produces an ROC curve for a WHtR-based treatment target. Comparing the area under the curve (AUC) for the WHtR ROC curve with the AUC for the BMI ROC curve allows us to determine which of these adiposity measures can better deliver a treatment target without focusing on a specific BMI or WHtR value, as the AUC provides an estimate of the overall potential of a measure to separate patients who have reached low risk from those who have not reached low risk.


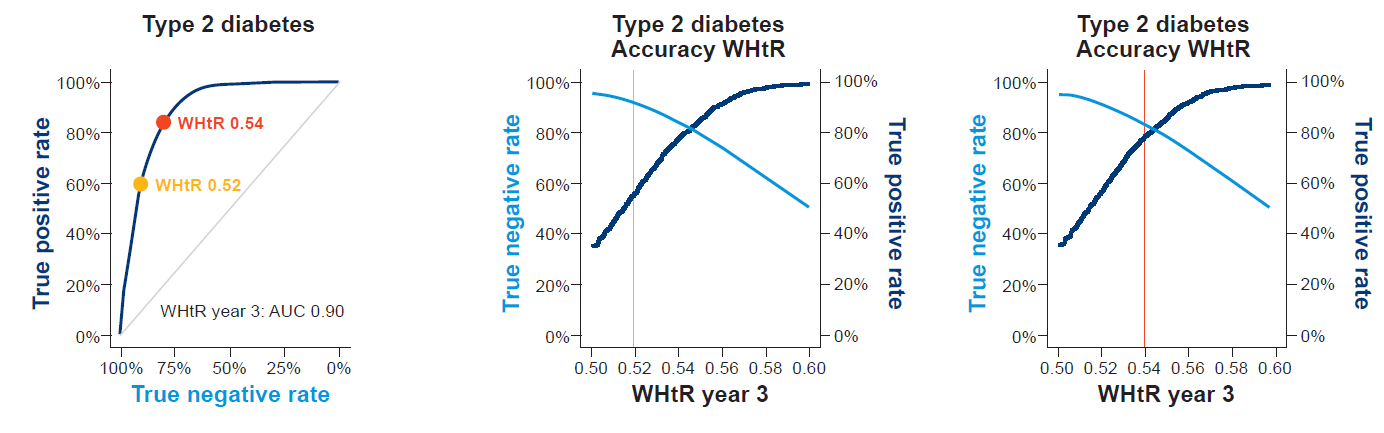


AUC, area under the curve; WHtR, waist–height ratio.

The ideal treatment target is a value that achieves 100% TPR and 100% TNR, although such a value does not exist in reality. TPR and TNR are interdependent, and as we move from the lowest to the highest WHtR value, the TPR/TNR ratio transitions from 0:100 to 100:0. In the figure above, the two rightmost graphs present the data points from the ROC curve differently: WHtR values are plotted on the x-axis, and separate lines for TPR and TNR are drawn across the WHtR range. A practical choice for a WHtR-based treatment target, indicating whether the low-risk state has been reached, would be the point where the two lines intersect. In the example above, this intersection occurs slightly above a WHtR of 0.54.

## Table S1. SNOMED CT codes used to identify unintentional weight loss

| SNOMED CT term | SNOMED CT concept ID |
| --- | --- |
| Abnormal weight loss | 267024001 |
| Abnormal weight loss – symptom | 267024001 |
| Abnormal loss of weight | 267024001 |
| Complaining of weight loss | 198511000000103 |
| Unintentional weight loss | 448765001 |
| Muscle wasting | 88092000 |
| Unexplained/progressive weight loss | 960561000006106 |
| O/E – quadriceps muscle wasting | 275851002 |
| Unexplained weight loss | 422868009 |
| HIV disease resulting in wasting syndrome | 422003001 |

HIV, human immunodeficiency virus; ID, identification data; SNOMED CT, Systematized Nomenclature of Medicine Clinical Terms.

## Table S2. Diagnosis codes used to identify obesity-related complications

|  | Diagnosis code | |
| --- | --- | --- |
|  | **CPRD data (SNOMED CT)** | **HES data (ICD-10)** |
| Type 2 diabetes | 170746002, 170745003, 314194001, 313436004, 420715001, 237599002, 44054006, 421986006, 422034002, 713703005, 421750000, 421326000, 421779007, 190389009, 314904008, 422014003, 443694000, 422099009, 420279001, 395204000, 422166005, 420756003, 314902007, 719216001, 190388001, 314903002, 199230006, 24481000000101, 421631007, 190331003, 420436000, 713706002 | E11, E11.0, E11.1, E11.2, E11.3, E11.4, E11.5, E11.6, E11.7, E11.8, E11.9 |
| Hypertension | 59621000, 185719002, 275944005, 24184005, 185720008, 270440008, 716771000000106, 185723005, 1201005, 185721007, 713641000000103, 185264001, 716821000000100, 170577003, 302192008, 697930002, 185716009, 185722000, 170578008, 473225006, 390925005, 38341003, 56218007, 170586008, 846371000000103, 736286003, 790031000006103, 717311000000102, 715051000000108, 843821000000102, 185718005, 78975002, 183856001, 908631000000108, 713661000000102, 170581003, 401117004, 38481006, 909441000006102, 48146000, 401047000, 908651000000101, 810981000000107, 70272006, 845891000000103, 843841000000109, 123799005, 194788005, 73410007, 194791005, 64715009, 54225002, 31992008, 194785008, 89242004, 194780003, 77970009, 36315003, 443482000 | I10, I11, I11.0, I11.9, I12, I12.0, I12.9, I13, I13.0, I13.1, I13.2, I13.9, I15, I15.0, I15.1, I15.2, I15.8, I15.9 |
| Hip/knee osteoarthritis | 201819000, 239872002, 430698003, 637091000000105, 52734007, 609588000, 19063003, 450521003, 396275006, 28181007, 179344006, 392238003, 909231000006108, 179319003, 265158005, 179304004, 239501000, 909221000006105, 179402001, 386649003, 201835007, 267891004, 183992003, 179342005, 765161000000106, 47458005, 265172001, 314489006, 179351002, 179407007, 307817008, 179403006, 310640003, 265157000, 179306002, 314491003, 239499008, 179326003, 179408002, 450813004, 265170009, 267890003, 280460009, 307814001, 738066004, 179412008, 307819006, 201841000, 762052005, 449039004, 179413003, 179405004, 280462001, 265160007, 450818008, 309458002, 239873007, 587231000000100, 323311000119107, 425443008, 179410000, 323291000119108 |  |
| ASCVD | 230690007, 400047006, 63491006, 57054005, 401303003, 432504007, 394659003, 36969009, 230691006, 73795002, 4557003, 274100004, 233819005, 195216008, 195217004, 194828000, 233817007, 175715003, 95460007, 307766002, 685631000000102, 307767006, 68466008, 194842008, 54329005, 71444005, 252094004, 175612005, 405469008, 3546002, 194843003, 195302000, 70211005, 300995000, 266254007, 1386000, 233958001, 16589005, 301755001, 307408003, 413838009, 203741000000101, 314188009, 22298006, 175036008, 230698000, 65547006, 70422006, 75038005, 194823009, 736968006, 413102000, 372070002, 175716002, 195213000, 195212005, 164861001, 11101003, 408546009, 75543006, 225566008, 85053006, 72092001, 175615007, 111298007, 195303005, 314187004, 20059004, 164867002, 233838001, 195254008, 62695002, 233961000, 164625009, 736966005, 195318006, 81817003, 50807007, 195323006, 314189001, 195168007, 76593002, 195189003, 414089002, 312688006, 233821000, 398274000, 58612006, 736969003, 413844008, 175689005, 233843008, 307140009, 300920004, 195185009, 399957001, 591161000000105, 29843007, 233384002, 195324000, 266253001, 52981000006108, 7713009, 195339004, 21631000119105, 175685004, 175704008, 231231000000107, 233823002, 194849004, 164868007, 388997005, 397193006, 230699008, 955491000000106, 443502000, 266257000, 233317001, 195190007, 164869004, 14201006, 314116003, 736967001, 53741008, 426093007, 59021001, 310357009, 315026000, 312227009, 195186005, 125081000119106, 233959009, 230716006, 68109007, 1823971000006102, 95457000, 78569004, 302909007, 277286006, 195341003, 194856005, 164865005, 44662004, 713412006, 175279003, 195180004, 1755008, 401314000, 719678003, 129574000, 64662007, 2008601000006100, 405376002, 230700009, 175071008, 232717009, 79009004, 405477007, 112828007, 164870003, 31413008, 1573101000006108, 304914007, 236371000000109, 164871004, 38716007, 307800001, 87343002, 232722009, 405557003, 95691008, 51018000, 275059001, 195335005, 582911000000104, 429639007, 312611005, 233383008, 194821006, 175705009, 1823891000006109, 405463009, 175406006, 233885007, 266262004, 415070008, 736972005, 175718001, 232720001, 15990001, 450696008, 52035003, 175029007, 707813002, 275040003, 233310004, 237451000000109, 734299002, 842721000000102, 421895002, 713674006, 699245006, 175717006, 232726007, 275434003, 414545008, 736973000, 175464007, 195239002, 905551000006101, 116360008, 311793000, 232721002 | G45, G45.0, G45.1, G45.2, G45.3, G45.4, G45.8, G45.9, I20, I20.0, I20.1, I20.8, I20.9, I21, I21.0, I21.1, I21.2, I21.3, I21.4, I21.9, I22, I22.0, I22.1, I22.8, I22.9, I23, I23.0, I23.1, I23.2, I23.3, I23.4, I23.5, I23.6, I23.8, I24, I24.0, I24.1, I24.8, I24.9, I25, I25.0, I25.1, I25.2, I25.3, I25.4, I25.5, I25.6, I25.8, I25.9, I60, I60.0, I60.1, I60.2, I60.3, I60.4, I60.5, I60.6, I60.7, I60.8, I60.9, I61, I61.0, I61.1, I61.2, I61.3, I61.4, I61.5, I61.6, I61.8, I61.9, I62, I62.0, I62.1, I62.9, I63, I63.0, I63.1, I63.2, I63.3, I63.4, I63.5, I63.6, I63.8, I63.9, I64, I65, I65.0, I65.1, I65.2, I65.3, I65.8, I65.9, I66, I66.0, I66.1, I66.2, I66.3, I66.4, I66.8, I66.9, I67, I67.0, I67.1, I67.2, I67.3, I67.4, I67.5, I67.6, I67.7, I67.8, I67.9, I68, I68.0, I68.1, I68.2, I68.8, I69, I69.0, I69.1, I69.2, I69.3, I69.4, I69.8, I70, I70.0, I70.1, I70.2, I70.8, I70.9, I73.9, Z95.1, Z95.5 |

ASCVD, atherosclerotic cardiovascular disease; CPRD, Clinical Practice Research Datalink; HES, Hospital Episode Statistics; ICD-10, International Classification of Diseases, 10th Revision; SNOMED CT, Systematized Nomenclature of Medicine Clinical Terms.

## Table S3. Characteristics of the cohort used for the additional cox proportional hazards model analyses incorporating smoking status, socioeconomic status and race

|  | Total | Overweight | Obesity I | Obesity II | Obesity III |
| --- | --- | --- | --- | --- | --- |
| N | 32,026 | 12,954 | 10,400 | 5393 | 3279 |
| Women | 14,615 (45.6%) | 5191 (40.1%) | 4585 (44.1%) | 2855 (52.9%) | 1984 (60.5%) |
| Men | 17,411 (54.4%) | 7763 (59.9%) | 5815 (55.9%) | 2538 (47.1%) | 1295 (39.5%) |
| Baseline characteristics, median (IQR) | | | | | |
| Age at index date, years | 51 (44–56) | 50 (44–56) | 51 (45–56) | 50 (44–55) | 50 (43–55) |
| BMI year 1, kg/m^2^ | 31.2 (28.1–35.4) | 27.5 (26.4–28.7) | 32.1 (31.0–33.4) | 37.0 (35.9–38.3) | 43.3 (41.4–46.2) |
| BMI year 4, kg/m^2^ | 31.3 (28.2–35.5) | 27.8 (26.3–29.2) | 32.2 (30.7–33.9) | 36.9 (35.3–38.7) | 42.9 (40.5–46.0) |
| BMI change, % | 0.1 (−3.3–3.7) | 0.6 (−2.5–4.2) | 0.1 (−3.3–3.7) | -0.3 (−4.0–3.0) | -1.2 (−5.4–2.8) |
| WHtR year 1 | 0.61 (0.56–0.67) | 0.56 (0.53–0.59) | 0.62 (0.59–0.65) | 0.68 (0.65–0.71) | 0.76 (0.72–0.80) |
| WHtR year 4 | 0.61 (0.56–0.68) | 0.56 (0.53–0.60) | 0.62 (0.59–0.66) | 0.68 (0.65–0.72) | 0.76 (0.71–0.80) |
| WHtR change, % | 0.3 (−3.1–4.4) | 0.5 (−2.6–4.7) | 0.4 (−3.0–4.5) | 0.1 (−3.5–3.9) | 0.0 (−4.1–3.6) |
| Follow-up, years | 8.1 (6.1–9.9) | 8.0 (6.1–9.7) | 8.2 (6.2–9.9) | 8.3 (6.1–10.0) | 8.1 (6.0–9.9) |
| Smoking status | | | | | |
| Current | 4596 (14.4%) | 1948 (15.0%) | 1477 (14.2%) | 729 (13.5%) | 442 (13.5%) |
| Ex | 12,746 (39.8%) | 4836 (37.3%) | 4223 (40.6%) | 2278 (42.2%) | 1409 (43.0%) |
| Never | 13,074 (40.8%) | 5517 (42.6%) | 4191 (40.3%) | 2118 (39.3%) | 1248 (38.1%) |
| Unknown | 1610 (5.0%) | 653 (5.0%) | 509 (4.9%) | 268 (5.0%) | 180 (5.5%) |

| Weight-loss interventions during baseline period | | | | | |
| --- | --- | --- | --- | --- | --- |
| Weight-loss drugs | 1984 (6.2%) | 182 (1.4%) | 576 (5.5%) | 621 (11.5%) | 605 (18.5%) |
| Bariatric surgery | 127 (0.4%) | 3 (0.0%) | 9 (0.1%) | 23 (0.4%) | 92 (2.8%) |
| Race |  |  |  |  |  |
| White | 25,426 (79.4%) | 9620 (74.3%) | 8310 (79.9%) | 4572 (84.8%) | 2924 (89.2%) |
| Asian | 4648 (14.5%) | 2511 (19.4%) | 1429 (13.7%) | 524 (9.7%) | 184 (5.6%) |
| Black | 1952 (6.1%) | 823 (6.4%) | 661 (6.4%) | 297 (5.5%) | 171 (5.2%) |
| Patient residence area socioeconomic status | | | | | |
| IMD 1 | 4985 (15.6%) | 2161 (16.7%) | 1676 (16.1%) | 763 (14.1%) | 385 (11.7%) |
| IMD 2 | 5342 (16.7%) | 2235 (17.3%) | 1758 (16.9%) | 858 (15.9%) | 491 (15.0%) |
| IMD 3 | 5625 (17.6%) | 2264 (17.5%) | 1830 (17.6%) | 942 (17.5%) | 589 (18.0%) |
| IMD 4 | 7493 (23.4%) | 3000 (23.2%) | 2371 (22.8%) | 1277 (23.7%) | 845 (25.8%) |
| IMD 5 | 8581 (26.8%) | 3294 (25.4%) | 2765 (26.6%) | 1553 (28.8%) | 969 (29.6%) |
| ORCs at start of follow-up | | | | | |
| Type 2 diabetes | 15,128 (47.2%) | 4555 (35.2%) | 5118 (49.2%) | 3274 (60.7%) | 2181 (66.5%) |
| Hypertension | 18,378 (57.4%) | 6280 (48.5%) | 6223 (59.8%) | 3574 (66.3%) | 2301 (70.2%) |
| Dyslipidemia | 15,164 (47.3%) | 5417 (41.8%) | 5174 (49.8%) | 2873 (53.3%) | 1700 (51.8%) |
| Hip/knee osteoarthritis | 2953 (9.2%) | 768 (5.9%) | 990 (9.5%) | 701 (13.0%) | 494 (15.1%) |
| ASCVD | 4449 (13.9%) | 1658 (12.8%) | 1519 (14.6%) | 793 (14.7%) | 479 (14.6%) |

| Diagnoses during follow-up per 1000 patient-years | | | | | |
| --- | --- | --- | --- | --- | --- |
| Type 2 diabetes | 18.1 | 11.9 | 20.3 | 30.3 | 34.6 |
| Hypertension | 46.4 | 38.0 | 50.5 | 59.7 | 66.4 |
| Hip/knee osteoarthritis | 14.8 | 10.6 | 16.0 | 18.4 | 24.2 |
| ASCVD | 13.7 | 12.0 | 14.4 | 15.6 | 15.0 |

Overweight, BMI 25 to <30 kg/m^2^; Obesity I, BMI 30 to <35 kg/m^2^; Obesity II, 35 to <40 kg/m^2^; Obesity III, ≥40 kg/m^2^.

ASCVD, atherosclerotic cardiovascular disease; BMI, body mass index; IMD, Index of Multiple Deprivation; IQR, interquartile range; ORC, obesity-related complication; WHtR, waist–height ratio.

## Table S4. Characteristics of the reference cohort used to derive the 10-year low obesity-related complication risk

|  | Total | Normal weight | Overweight |
| --- | --- | --- | --- |
| N | 318,178 | 136,785 | 181,393 |
| Women | 163,420 (51.4%) | 83,981 (61.4%) | 79,439 (43.8%) |
| Men | 154,758 (48.6%) | 52,804 (38.6%) | 101,954 (56.2%) |
| Baseline characteristics, median (IQR) | | | |
| Index-date age, years | 49 (43–55) | 48 (42–54) | 49 (43–55) |
| Index-date BMI, kg/m^2^ | 25.6 (23.4–27.6) | 23.0 (21.6–24.1) | 27.3 (26.2–28.6) |
| Index-date WHtR | 0.52 (0.48–0.56) | 0.48 (0.45–0.51) | 0.55 (0.52–0.58) |
| Follow-up, years | 10.0 (8.1–11.7) | 9.9 (8.0–11.5) | 10.1 (8.3–11.8) |
| Comorbidities at start of follow-up | | | |
| Type 2 diabetes | 20,477 (6.4%) | 5077 (3.7%) | 15,400 (8.5%) |
| Hypertension | 45,771 (14.4%) | 12,892 (9.4%) | 32,879 (18.1%) |
| Hip/knee osteoarthritis | 8852 (2.8%) | 2798 (2.0%) | 6054 (3.3%) |
| ASCVD | 7965 (2.5%) | 2278 (1.7%) | 5687 (3.1%) |
| Diagnoses during follow-up per 1000 patient-years | | | |
| Type 2 diabetes | 4.8 | 2.3 | 6.8 |
| Hypertension | 20.5 | 14.6 | 25.7 |
| Hip/knee osteoarthritis | 7.2 | 5.3 | 8.6 |
| ASCVD | 4.7 | 3.7 | 5.5 |

ASCVD, atherosclerotic cardiovascular disease; BMI, body mass index; IQR, interquartile range; WHtR, waist–height ratio.

Table S5. True positive and true negative rates for potential BMI and WHtR targets, alone and combined, as indicators of a low absolute 10-year risk of ORCs after weight loss in the weight-change cohort (*N* = 45,899)

| ORC | BMI 27 kg/m^2^ | | WHtR 0.53 | | BMI 27 kg/m^2^ and WHtR 0.53 | |
| --- | --- | --- | --- | --- | --- | --- |
|  | **TPR** | **TNR** | **TPR** | **TNR** | **TPR** | **TNR** |
| Type 2 diabetes | 79.7% | 86.5% | 95.4% | 91.4% | 75.9% | 96.3% |
| Hypertension | 95.4% | 80.9% | 99.4% | 84.7% | 94.7% | 90.9% |
| Hip/knee osteoarthritis | 89.0% | 87.0% | 55.5% | 90.1% | 53.2% | 94.6% |
| ASCVD | 51.0% | 85.5% | 62.2% | 90.1% | 41.5% | 94.0% |

TNR is the proportion of patients with a risk higher than the low reference risk and above the target value. TPR is the ratio of all patients with a risk equal to or lower than the reference risk and who have a value for the adiposity measure below target. ASCVD, atherosclerotic cardiovascular disease; BMI, body mass index; ORC, obesity-related complication; TNR, true negative rate; TPR, true positive rate; WHtR, waist–height ratio.

## Table S6. Characteristics of the baseline cohort and a cohort based on individuals with a BMI record only, no waist (or WHtR) measurement, during 2010 and 2014.

|  | Baseline cohort | No WHtR cohort |
| --- | --- | --- |
| N | 499,813 | 3,093,991 |
| Women | 233,454 (46.7%) | 1,573,358 (50.9%) |
| Men | 266,359 (53.3%) | 1,520,633 (49.1%) |
| Baseline characteristics, median (IQR) | | |
| Age (years) | 48 (41, 54) | 42 (31, 51) |
| BMI (kg/m^2^) | 29.8 (27.2, 33.7) | 28.9 (26.6, 32.7) |
| Follow-up (years) | 9.9 (6.4, 11.9) | 9.7 (4.6, 12.1) |
| Comorbidities at start of follow-up | | |
| Type 2 diabetes | 76,433 (15.3%) | 173,496 (5.6%) |
| Hypertension | 131,320 (26.3%) | 543,195 (17.6%) |
| Dyslipidemia | 81,818 (16.4%) | 279,034 (9.0%) |
| Hip/knee osteoarthritis | 22,119 (4.4%) | 80,671 (2.6%) |
| ASCVD | 26,652 (5.3%) | 90,150 (2.9%) |
| Diagnoses during follow-up per 1,000 patient-years | | |
| Type 2 diabetes | 12.9 | 8.8 |
| Hypertension | 33.7 | 23.1 |
| Hip/knee osteoarthritis | 11.0 | 7.8 |
| ASCVD | 8.6 | 4.5 |

ASCVD, atherosclerotic cardiovascular disease; BMI, body mass index; IQR, interquartile range; WHtR, waist–height ratio.

## Figure S1. Study designs


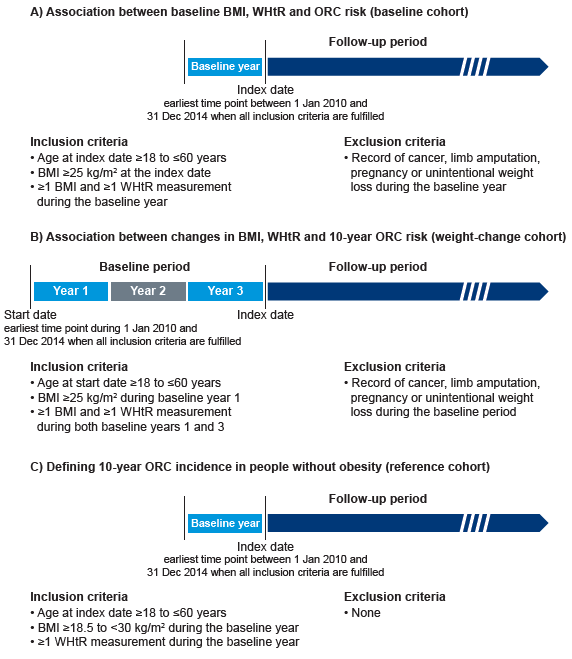


BMI, body mass index; ORC, obesity-related complication; WHtR, waist–height ratio.

## Figure S2. Participant disposition


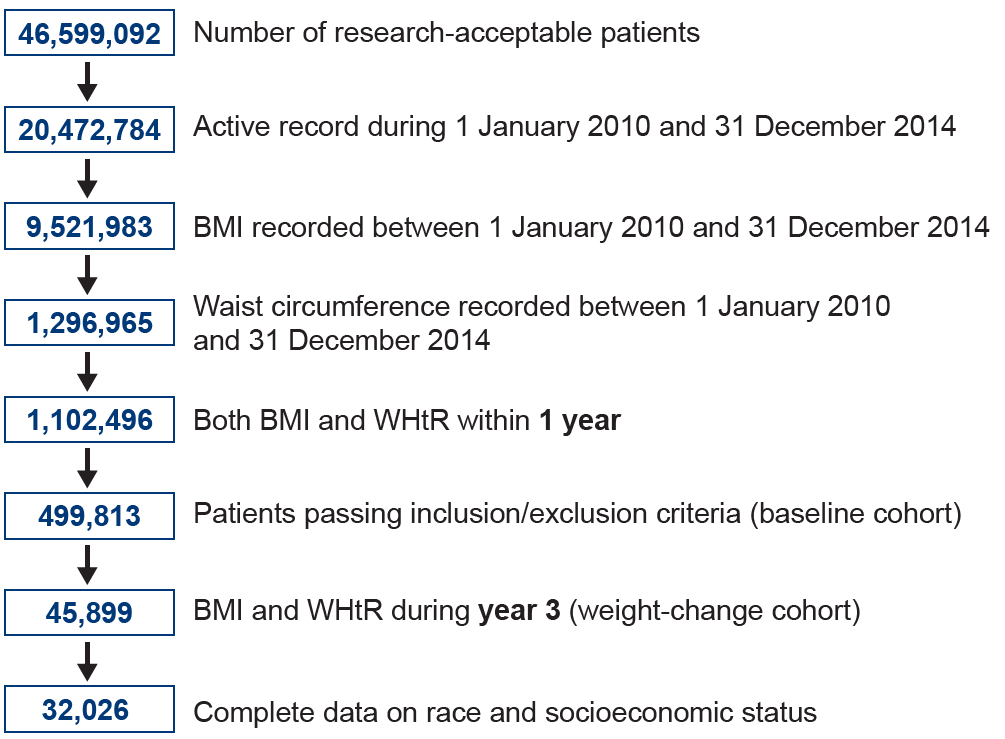


BMI, body mass index; WHtR, waist–height ratio.

## Figure S3. Association between baseline characteristics and obesity-related complication hazard ratio in the baseline cohort (*N* = 499,813)

##
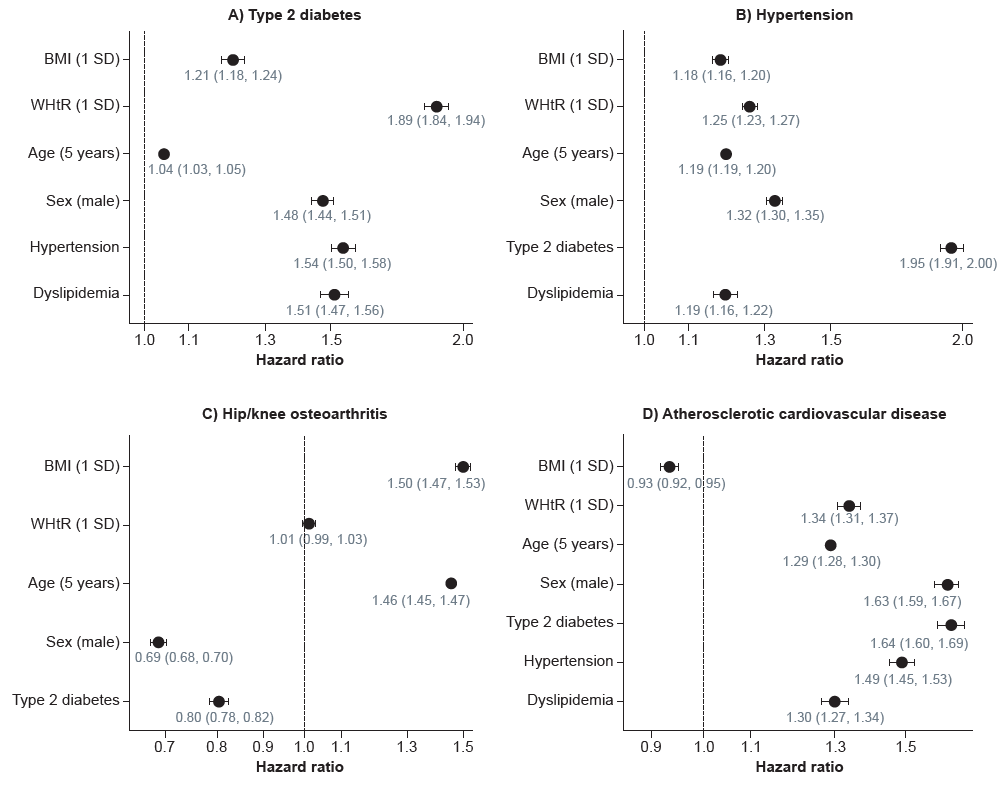
Association between measures and the risk of developing type 2 diabetes, hypertension, hip/knee osteoarthritis or ASCVD were calculated using a Cox proportion hazard regression analysis, with the different measures as covariates at baseline. The models included quadratic interaction terms of anthropometric measures. For BMI, 1 SD corresponds to approximately 5 kg/m2. For WHtR, 1 SD corresponds to approximately 0.08. For age, ‘5 years’ corresponds to an increase of 5 years in age. Data labels signify hazard ratio (95% confidence interval). ASCVD, atherosclerotic cardiovascular disease; BMI, body mass index; SD, standard deviation; WHtR, waist–height ratio.

## Figure S4. Changes in BMI and WHtR during follow-up in the cohort used for analyses of the associations between weight loss and obesity-related complication risk (*N* = 45,899)


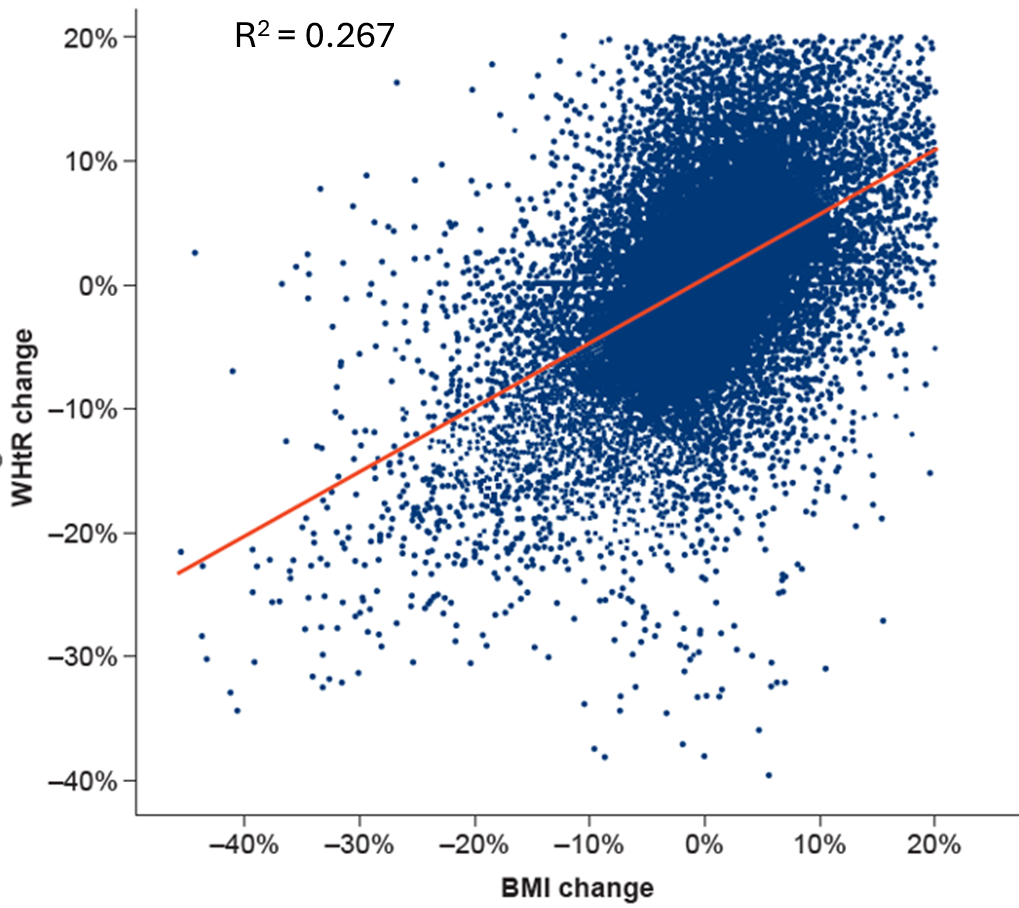


Each data point corresponds to an observed change. The red line represents the line of regression. The R^2^ value measures how well the linear regression model predicts an outcome (dependent variable) on a scale of 0–1 (0, does not predict the outcome; 0-1, partially predicts the outcome; 1, perfectly predicts the outcome). BMI, body mass index; R^2^, coefficient of determination squared; WHtR, waist–height ratio.

## Figure S5. Pattern of 10-year risk of obesity-related complications by BMI and WHtR change for women aged 50 years with BMI 33 kg/m^2^ and WHtR 0.61 in the weight-change cohort


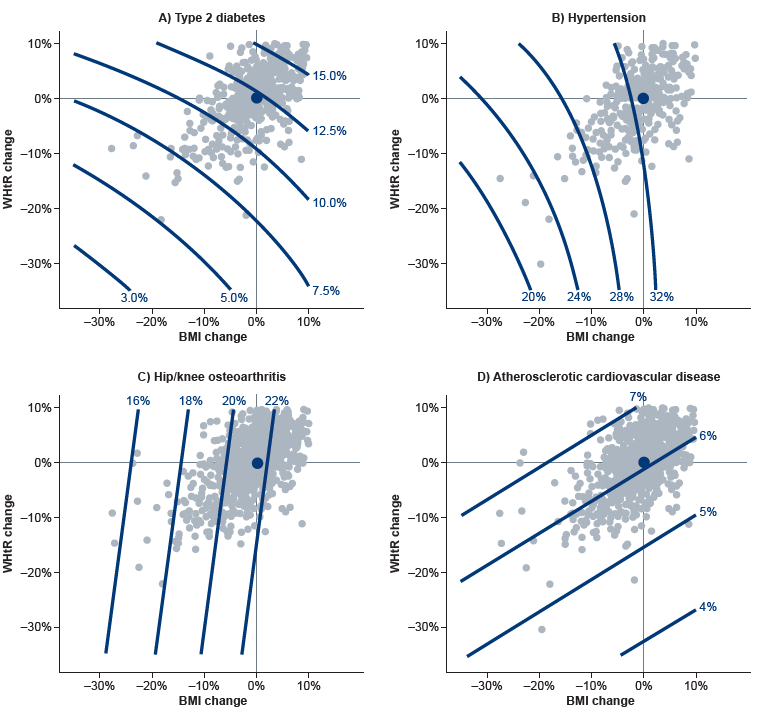


BMI, body mass index; WHtR, waist–height ratio.

## Figure S6. Contribution of the covariates in the additional Cox proportional hazard model for the weight-change cohort, including smoking status, race and socioeconomic status (*N* = 32,026)


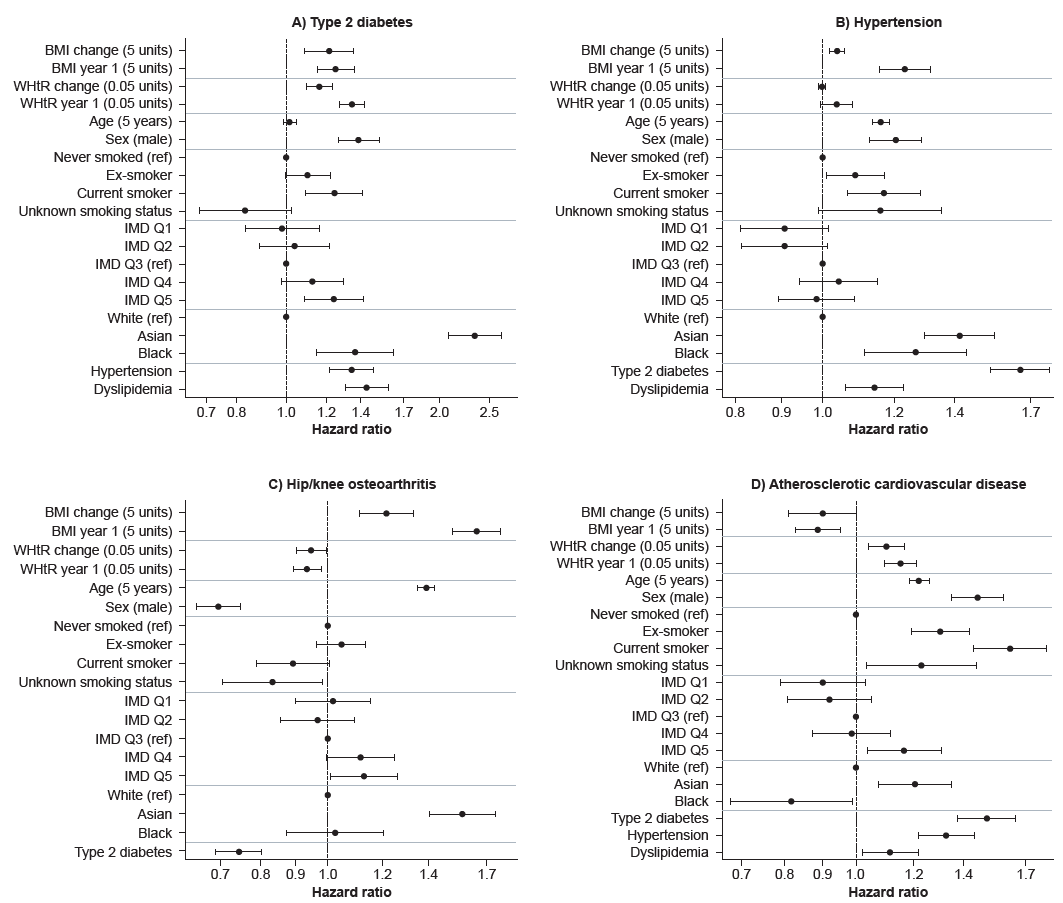


Association between measures and the risk of developing type 2 diabetes, hypertension, hip/knee osteoarthritis, or atherosclerotic cardiovascular disease were calculated using a Cox proportion hazard regression analysis, with the different measures as covariates at baseline, conducted among the subset of individuals who had complete data on race and socioeconomic status. Error bars are 95% confidence intervals. BMI, body mass index; IMD, Index of Multiple Deprivation; WHtR, waist–height ratio.

## Figure S7. Sex-specific 10-year incidence of obesity-related complications by age for the reference cohort of individuals without obesity (*N* = 318,178)


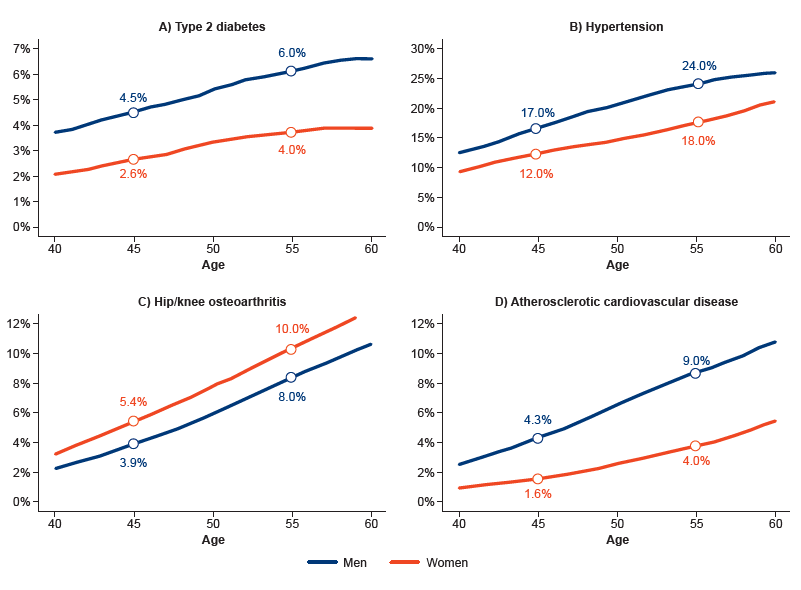


Data are shown for patients aged 40–60 years only, for illustrative purposes, as the incidence of ORCs in the reference cohort
was low. ORC, obesity-related complication.

## Figure S8. Accuracy for BMI and WHtR as treatment targets for achieving a low reference 10-year risk of obesity-related complications in the weight-change cohort (*N* = 45,899)


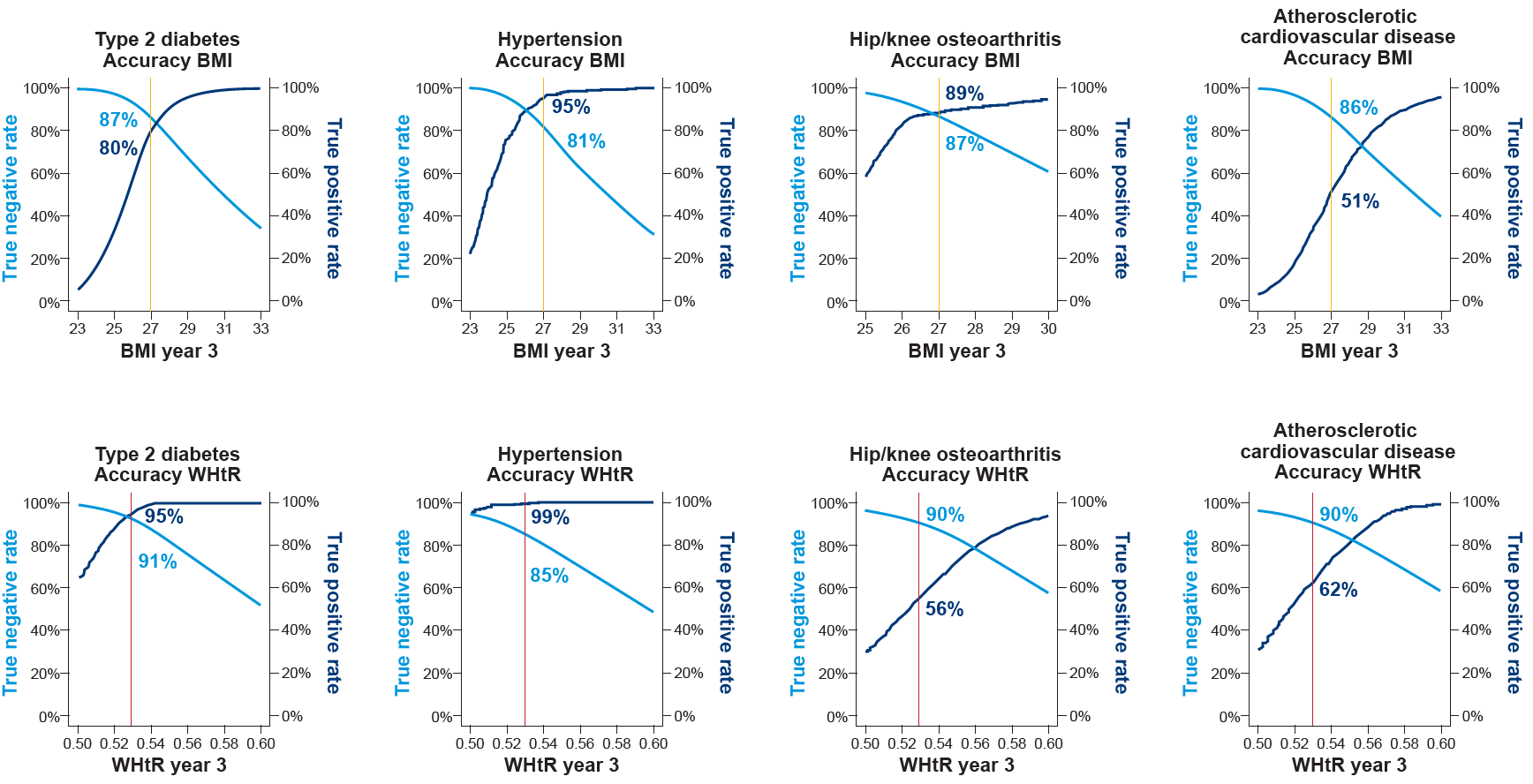


The TPR and TNR for different BMI and WHtR values. The yellow line represents BMI 27 kg/m^2^ as the treatment target, and the red line represents WHtR 0.53 as the treatment target. BMI, body mass index; TNR, true negative rate; TPR, true positive rate; WHtR, waist–height ratio.
